# Characteristics of autonomic dysfunction in neuronal intranuclear inclusion disease

**DOI:** 10.3389/fneur.2023.1168904

**Published:** 2023-06-14

**Authors:** Lu Zhou, Yun Tian, Sizhe Zhang, Bin Jiao, Xinxin Liao, Yafang Zhou, Qiao Xiao, Jin Xue, Ranhui Duan, Beisha Tang, Lu Shen

**Affiliations:** ^1^Department of Neurology, Xiangya Hospital, Central South University, Changsha, Hunan, China; ^2^Department of Geriatrics, Xiangya Hospital, Central South University, Changsha, Hunan, China; ^3^National Clinical Research Center for Geriatric Disorders, Central South University, Changsha, Hunan, China; ^4^Engineering Research Center of Hunan Province in Cognitive Impairment Disorders, Central South University, Changsha, Hunan, China; ^5^School of Life Sciences, Central South University, Changsha, Hunan, China; ^6^Key Laboratory of Hunan Province in Neurodegenerative Disorders, Central South University, Changsha, Hunan, China; ^7^Hunan International Scientific and Technological Cooperation Base of Neurodegenerative and Neurogenetic Diseases, Changsha, Hunan, China; ^8^Key Laboratory of Organ Injury, Aging and Regenerative Medicine of Hunan Province, Changsha, China

**Keywords:** neuronal intranuclear inclusion disease, autonomic dysfunction, SCOPA-AUT, assessment, autonomic symptoms

## Abstract

**Background:**

This study aimed to investigate the features of autonomic dysfunction (AutD) in a large cohort of patients with neuronal intranuclear inclusion disease (NIID).

**Methods:**

A total of 122 patients with NIID and 122 controls were enrolled. All participants completed the Scales for Outcomes in Parkinson’s Disease-Autonomic Questionnaire (SCOPA-AUT) and genetic screening for GGC expanded repeats within the *NOTCH2NLC* gene. All patients underwent neuropsychological and clinical assessments. SCOPA-AUT was performed to compare AutD between patients and controls. The associations between AutD and disease-related characteristics of NIID were studied.

**Results:**

94.26% of patients had AutD. Compared with controls, patients had more severe AutD in total SCOPA-AUT, gastrointestinal, urinary, cardiovascular, thermoregulatory, pupillomotor and sexual domains (all *p* < 0.05). The area under the curve (AUC) value for the total SCOPA-AUT (AUC = 0.846, sensitivity = 69.7%, specificity = 85.2%, cutoff value = 4.5) was high in differentiating AtuD of patients with NIID from controls. The total SCOPA-AUT was significantly and positively associated with age (*r* = 0.185, *p* = 0.041), disease duration (*r* = 0.207, *p* = 0.022), Neuropsychiatric Inventory (NPI) (*r* = 0.446, *p* < 0.01), and Activities of Daily Living (ADL) (*r* = 0.390, *p* < 0.01). Patients with onset-of-AutD had higher SCOPA-AUT scores than patients without onset-of-AutD (*p* < 0.001), especially in the urinary system (*p* < 0.001) and male sexual dysfunction (*p* < 0.05).

**Conclusion:**

SCOPA-AUT can be used as a diagnostic and quantitative tool for autonomic dysfunction in NIID. The high prevalence of AutD in patients suggests that NIID diagnosis should be considered in patients with AutD, especially in those with unexplained AutD alone. AutD in patients is related to age, disease duration, impairment of daily living ability, and psychiatric symptoms.

## Introduction

Neuronal intranuclear inclusion disease (NIID) is a rare neurodegenerative disease that is characterized by intranuclear inclusions in the central and peripheral nervous systems and in other organs (1–3). In 2019, an expansion of GGC repeats located in the 5′ UTR of the *NOTCH2NLC* gene was found to be associated with individuals with NIID, mostly in people of Asian origin (4–7). Clinically, NIID symptoms are highly heterogeneous, with various dysfunctions of the central and peripheral nervous systems, including progressive dementia and cognitive impairment, parkinsonism, stroke-like episodes, encephalitic episodes, cerebellar ataxia, sensory disturbance, peripheral neuropathy, and autonomic dysfunction (3, 7–9).

Many patients with NIID, especially those of increased age, usually show autonomic dysfunction (AutD), such as urinary incontinence, miosis, vomiting, gastrointestinal dysfunction, orthostatic hypotension, arrhythmia, and sexual dysfunction (3, 7, 8, 10–12). Sone et al. (3) reported that the prevalence of bladder dysfunction (33.3% in sporadic cases and 62.5% in familial cases), miosis (94.4% in sporadic cases and 63.6% in familial cases), vomiting (15.8% in sporadic cases and 31.6% in familial cases), and syncope (8.1% in sporadic cases and 0% in familial cases) was high in a Japanese cohort. Our group has also found that AutD was frequently observed in 64.0% (158/247) of patients with NIID in a large Chinese cohort, and bladder dysfunction (48.4%), miosis (20.8%), emesis (14.6%), and orthostatic hypotension (13.5%) were the most common AutDs in that study (13). Interestingly, AutD has been observed in some patients as their only symptom for many years, indicating that AutD may be an optional diagnostic biomarker to identify NIID (14). However, the prevalence and detailed clinical features of AutD in patients with NIID have rarely been studied.

The Scales for Outcomes in Parkinson’s Disease-Autonomic Questionnaire (SCOPA-AUT) (15) has 23 items in six regions. It is a reliable and valid questionnaire that encompasses the full spectrum of autonomic problems (16) and is widely used to assess autonomic symptoms in patients with Parkinson’s disease (PD) (17, 18) and other neurodegenerative diseases (19–21).

This study aimed to use SCOPA-AUT to investigate the prevalence and clinical characteristics of AutD in patients with NIID and the correlations between AutD and other NIID symptoms in a large cohort.

## Materials and methods

### Participants

A total of 122 patients with genetically confirmed NIID were enrolled between January 2018 and December 2021. All the patients were evaluated by at least two neurologists. NIID was diagnosed based on clinical features and abnormal GGC repeats (>65) within the *NOTCH2NLC*. One hundred and twenty-two age- and sex-matched healthy controls were enrolled, and genetic tests were performed to exclude NIID. This study was approved by the Ethics Committee of the Xiangya Hospital, Central South University. Written informed consent was obtained from all the participants at enrollment.

### Clinical assessment and genetic testing

Clinical manifestation data were collected during the clinical examination interviews. The SCOPA-AUT was used to assess AutD in all participants. For patients, the Mini-Mental State Examination (MMSE) (22), Montreal Cognitive Assessment Scale (MoCA) (23) and Frontal Assessment Battery (FAB) (24) were used to screen for cognitive impairments; the Neuropsychiatric Inventory (NPI) (25) was used to assess mental problems; and the Activities of Daily Living scale (ADL) (26) was used to assess activities of daily living. Repeat-primed PCR (RP-PCR) and GC-rich PCR (GC-PCR) assays were performed to confirm GGC expanded repeats within *NOTCH2NLC* as previously described (7).

### Definition of AutD

AutD was defined as a score ≥1 in SCOPA-AUT. A score of zero was regarded as normal autonomic function.

### Statistical analyses

Descriptive summaries are reported as medians (interquartile range, IQR) for continuous variables and percentages for categorical variables. Mann–Whitney *U* tests and chi-square tests were performed for variance analysis. Multivariate linear regression analyses were used to adjust for sex and/or age. A receiver operating characteristic (ROC) curve was generated to assess the prediction accuracy of the SCOPA-AUT for NIID. Spearman’s rank test was used for correlation analysis. Differences with *p* < 0.05 were considered statistically significant. Statistical analyses were performed using SPSS version 26.0 (IBM SPSS, Inc., Chicago, IL, United States).

## Results

### Demographic characteristics

A total of 122 patients with NIID and 122 control participants were enrolled in this study. Patients and controls had similar sex proportion (male: female total ratio of 58/64 vs. 55/67, *p* = 0.700). The median (IQR) age was 65 (59–69) years in the patients with NIID and 65 (63–68) years in the control participants (*p* = 0.434). The median expansion of *NOTCH2NLC* was 116.5 (100.5–139.5) in the NIID group and 19 (16–21) in the control group (*p* < 0.001). Patients with NIID had a median (IQR) disease duration of 5 (2–10) years and a median (IQR) age at onset of 60 (52–64) years. The manifestations of patients with NIID were summarized as five symptom categories: cognitive impairment (49.18%), muscle weakness (34.43%), movement disorders(56.56%), paroxysmal symptom (including disturbance of consciousness, encephalitic episodes, stroke-like episodes, generalized convulsions and chronic headache, 72.95%), and autonomic dysfunction (70.49%). 86/122 patients with NIID complained of autonomic dysfunction. 18 patients had undergone cystostomy or long-term indwelling catheter insertion. Detailed information is shown in Table 1.

**Table 1:** Demographics and clinical characteristics of participants.

	Patients (*n* = 122)	Controls (*n* = 122)	*p*-value
Age (median, IQR)	65 (59–69)	65 (63–68)	0.434^b^
Gender ratio (male/female)	58/64	55/67	0.700^a^
Onset age (median, IQR)	60 (52–64)	–	–
Disease duration (median, IQR)	5 (2–10)	–	–
GGC repeat sizes (median, IQR)	116.5 (100.5–139.5)	19.0 (16.0–21.0)	<0.001^b^
Manifestations, *n* (%)			
Cognitive impairment	60 (49.18)	–	–
Muscle weakness	42 (34.43)	–	–
Movement disorders	69 (56.56)	–	–
Paroxysmal symptom	89 (72.95)	–	–
Autonomic dysfunction	86 (70.49)	–	–

a Chi-square test.

b Mann–Whitney test.

### SCOPA-AUT scores in patients with NIID and control participants

In patients with NIID, 94.26% (115/122) had AutD, and the most frequently affected domain in NIID with AutD was the urinary (98/122, 80.33%), followed by the gastrointestinal (89/122, 72.95%), thermoregulatory (56/122, 45.9%), cardiovascular (54/122, 44.26%), pupillomotor dysfunction (31/122, 25.41%), and sexual (14/122, 11.48%) domains. The median SCOPA-AUT score was 11.00 (4.00–18.00). Compared with the control participants, patients with NIID displayed more severe AutD in the total SCOPA-AUT, gastrointestinal, urinary, cardiovascular, thermoregulatory, and pupillomotor domains (all *p* < 0.01). There were significant difference in the sexual domain (adjusted *p* = 0.043) and sexual domain of men (adjusted *p* = 0.010) between the NIID and control groups. The details are presented in Table 2. Given that females may tend to refuse admitting sexual problems on the questionnaire, the statistical analysis of the female sexual domain may be biased.

**Table 2:** Autonomic symptom severity and frequency (% with an item score ≥1) in the study population.

	Patients (*n* = 122)	Controls (*n* = 122)	*p*-value	Adjusted *p*-value
Total SCOPA-AUT score (median, IQR)	11.00 (4.00–18.00)	2.00 (0.00–3.25)	<0.001^b^	<0.001^c^
Gastrointestinal domain (median, IQR)	2.00 (0.00–4.25)	0.00 (0.00–1.00)	<0.001^b^	<0.001^c^
Swallowing/choking, *n* (%)	44 (36.07)	10 (8.20)	<0.001^a^	–
Sialorrhea, *n* (%)	28 (22.95)	5 (4.10)	<0.001^a^	–
Dysphagia, *n* (%)	19 (15.57)	4 (3.28)	0.001^a^	–
Early abdominal fullness, *n* (%)	27 (22.13)	9 (7.38)	0.001^a^	–
Constipation, *n* (%)	51 (41.80)	15 (12.30)	<0.001^a^	–
Straining for defecation, *n* (%)	51 (41.80)	17 (13.93)	<0.001^a^	–
Fecal incontinence, *n* (%)	13 (10.66)	0 (0.00)	<0.001^a^	–
Urinary domain (median, IQR)	4.00 (1.00–9.00)	1.00 (0.00–2.00)	<0.001^b^	<0.001^c^
Urgency, *n* (%)	50 (40.98)	11 (9.02)	<0.001^a^	–
Urinary incontinence *n* (%)	44 (36.07)	1 (0.82)	<0.001^a^	–
Incomplete emptying of bladder *n* (%)	60 (49.18)	10 (8.20)	<0.001^a^	–
Weak stream of urine, *n* (%)	50 (40.98)	11 (9.02)	<0.001^a^	–
Frequency of urinatior, *n* (%)	50 (40.98)	12 (9.84)	<0.001^a^	–
Nocturia, *n* (%)	83 (68.03)	53 (43.44)	<0.001^a^	–
Cardiovascular domain (median, IQR)	0.00 (0.00–2.00)	0.00 (0.00–0.00)	<0.001^b^	<0.001^c^
Lightheaded (standing up), *n* (%)	44 (36.07)	10 (8.20)	<0.001^a^	–
Lightheaded (standing for some time), *n* (%)	35 (28.69)	4 (3.28)	<0.001^a^	–
Syncope, *n* (%)	15 (12.30)	0 (0.00)	<0.001^a^	–
Thermoregulatory domain (median, IQR)	0.00 (0.00–3.00)	0.00 (0.00–0.25)	<0.001^b^	<0.001^c^
Hyperhidrosis (day), *n* (%)	26 (21.31)	11 (9.02)	0.007^a^	–
Hyperhidrosis (night), *n* (%)	24 (19.67)	2 (1.64)	<0.001^a^	–
Cold intolerance, *n* (%)	32 (26.23)	12 (9.84)	0.001^a^	–
Heat intolerance, *n* (%)	26 (21.31)	13 (10.66)	0.023^a^	–
Pupillomotor	0.00 (0.00–1.00)	0.00 (0.00–0.00)	<0.001^b^	0.014^c^
Oversensitive for bright light, *n* (%)	31 (25.41)	11 (9.02)	0.001^a^	–
Sexual domain (median, IQR)	0.00 (0.00–0.00)	0.00 (0.00–0.00)	0.555^b^	0.043^c^
Sexual domain: men (median, IQR)	0.00 (0.00–0.00)	0.00 (0.00–0.00)	0.309^b^	0.010^d^
Erection problem, *n* (%)	11 (18.97)	8 (14.55)	0.477^a^	–
Ejaculation problem, *n* (%)	10 (17.24)	8 (14.55)	0.636^a^	–
Sexual domain: women (median, IQR)	0.00 (0.00–0.00)	0.00 (0.00–0.00)	0.705^b^	0.611^d^
Vaginal lubrication dysfunction, *n* (%)	3 (4.69)	4 (5.97)	0.713^a^	–
Problem with orgasm, *n* (%)	2 (3.13)	4 (5.97)	0.414^a^	–

a Chi-square test.

b Mann–Whitney test.

c Adjusted by sex and age.

d Adjusted by age.

### Autonomic symptoms in patients with NIID and controls

The most frequently involved symptoms in patients with NIID were nocturia (68.03%), incomplete emptying of bladder (49.18%), constipation (41.80%), straining for defecation (40.98%), and urinary urgency (40.98%). The least frequently involved symptoms were problem with orgasm (3.13%), dysfunction of vaginal lubrication (4.69%), fecal incontinence (10.66%), syncope (12.30%), and dysphagia (15.57%). The largest differences between patients with NIID and control participants were the frequency of incomplete emptying of bladder (49.18% vs. 8.20%), urinary incontinence (36.07% vs. 0.82%), urinary urgency (40.98% vs. 9.02%), weak stream of urine (40.98% vs. 9.02%), and lightheadedness upon standing (36.07% vs. 8.20%) (Table 2). There were statistically significant differences (all *p* < 0.05) (Table 2) between patients with NIID and control participants in all symptoms, except sexual symptoms.

### Diagnostic value of SCOPA-AUT for AutD in NIID

**Figure 1:**
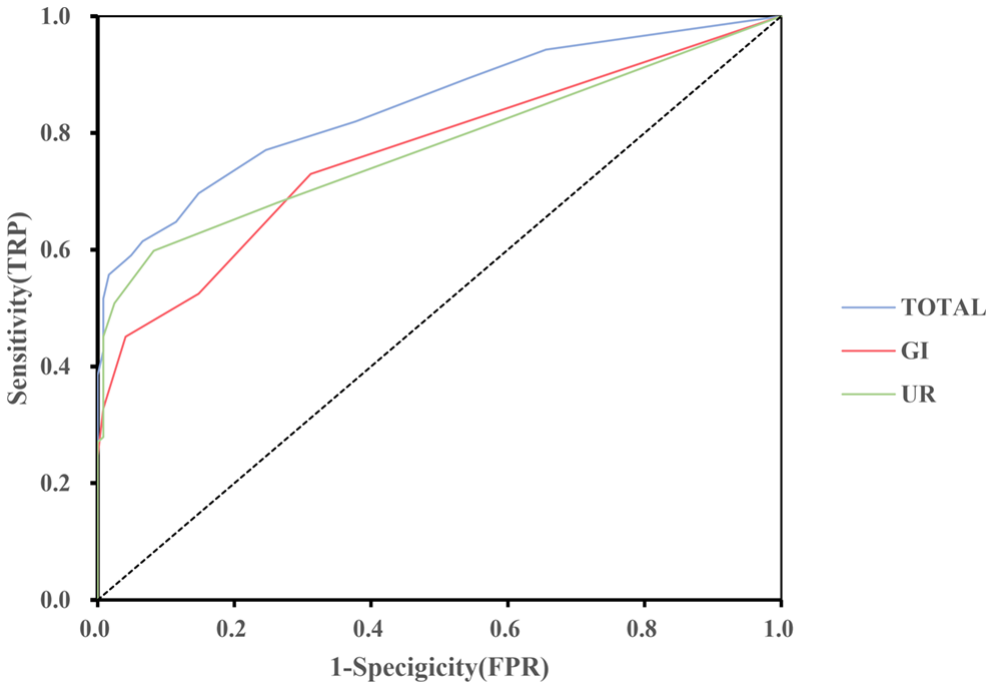
Receiver operating characteristic curve of the SCOPA-AUT and domains to diagnosis for AutD in NIID. GI, gastrointestinal functioning; UR, urinary functioning.

To detect the diagnostic accuracy of SCOPA-AUT for AutD in NIID, we performed ROC curve analysis. As presented in Table 3, the area under the curve (AUC) values were the highest for the total SCOPA-AUT (AUC = 0.846, sensitivity =69.7%, specificity = 85.2%, cutoff value = 4.5), gastrointestinal domain (AUC = 0.765, sensitivity = 73.0%, specificity = 68.9%, cutoff value = 0.5), and urinary domain (AUC = 0.776, sensitivity = 59.8%, specificity = 91.8%, cutoff value = 2.5). The ROC curves are shown in Figure 1. The AUC values for the other domains were <0.7. The results suggest that SCOPA-AUT, gastrointestinal and urinary symptoms, showed high diagnostic performance in differentiating AutD of patients with NIID from healthy controls and could be a diagnostic and quantitative tool for AutD in NIID.

**Table 3:** Total SCOPA-AUT score and domains for diagnosis of AutD in NIID (% with an item score ≥1).

	NIID patients (*n* = 122)	Controls (*n* = 122)	Se	Sp	Cutoff value	AUC	95% CI	*p*-value
Total	115 (94.3%)	80 (65.6%)	0.697	0.852	4.5	0.846	0.797–0.895	<0.001
GI	89 (73.0%)	38 (31.1%)	0.730	0.689	0.5	0.765	0.705–0.825	<0.001
UR	98 (80.3%)	67 (54.9%)	0.598	0.918	2.5	0.776	0.716–0.837	<0.001
CV	54 (44.3%)	10 (8.2%)	0.443	0.902	0.5	0.684	0.617–0.751	<0.001
TH	56 (45.9%)	30 (24.6%)	0.361	0.893	1.5	0.635	0.565–0.705	<0.001
PU	31 (25.4%)	11 (9.0%)	0.254	0.910	0.5	0.588	0.517–0.660	0.017
SX	14 (11.5%)	12 (9.8%)	0.082	0.902	2.5	0.512	0.439–0.584	0.752

GI, gastrointestinal functioning; UR, urinary functioning; CV, cardiovascular functioning; TH, thermoregulatory functioning; PU, pupillomotor functioning; SX, sexual functioning; Se, sensitivity; Sp, specificity; AUC, area under the curve; CI, confidence interval.

### Correlation between SCOPA-AUT and clinical factors of patients with NIID

The presence and severity of AutD may be affected by diverse clinical factors, as some autonomic phenotypes may occur simultaneously with other clinical symptoms. Spearman analysis was used to thoroughly investigate the correlation between SCOPA-AUT and other clinical manifestations. The total SCOPA-AUT score was significantly and positively associated with age (*r* = 0.185, *p* = 0.041), disease duration (*r* = 0.207, *p* = 0.022), NPI (*r* = 0.446, *p* < 0.01), and ADL (*r* = 0.390, *p* < 0.01) in patients with NIID. Meanwhile, the total SCOPA-AUT score was not correlated with age of onset, expanded repeat sizes within the *NOTCH2NLC* gene, MMSE, MoCA, or FAB (*p* > 0.05). The details are presented in Table 4.

**Table 4:** Correlation between SCOPA-AUT and NIID disease characteristics.

	*r*	*p*-value
Age	0.185	0.041
Age of onset	0.077	0.397
Disease duration	0.207	0.022
Expansion of *NOTCH2NLC*	−0.054	0.590
MMSE	−0.196	0.052
MoCA	−0.165	0.110
FAB	−0.193	0.069
NPI	0.446	<0.001
ADL	0.390	<0.001

### SCOPA-AUT differences between patients with or without the first onset of AutD

Eighteen of 122 (14.75%) patients with NIID had AutD as the first symptom, a condition referred to as onset-with-AutD NIID. All patients started with urinary symptoms of unknown origin, and the relevant symptomatic treatment was ineffective. In severe cases, cystostomy or long-term indwelling catheter insertion was performed before the appearance of other symptoms. The median SCOPA-AUT score of the patients with onset-with-AutD NIID was 19.00 (15.00–24.00). Other patients (85.25%) who did not present with AutD as their initial symptoms were referred to as patients with onset-without-AutD NIID. The median SCOPA-AUT score in this subgroup was 9.50 (3.00–16.00). Patients with onset-with-AutD had more severe SCOPA-AUT scores than patients with NIID in the onset-without-AutD group (*p* < 0.001), and the two groups also differed in urinary system (*p* < 0.001) and male sexual dysfunction (adjusted *p* = 0.026), while there were no differences in other domains. There were no differences in age (*p* = 0.894), sex (*p* = 0.821), age of onset (*p* = 0.734), duration of disease (*p* = 0.367), and expanded repeat sizes within *NOTCH2NLC* (*p* = 0.883) between the two groups. Detailed information is shown in Table 5.

**Table 5:** Difference between onset-without-AutD cases and onset-with-AutD cases.

	Onset-without-AutD cases (*n* = 104)	Onset-with-AutD cases (*n* = 18)	*p*-value	Adjusted *p*-value
Age (median, IQR)	65 (59–69)	65 (58–67)	0.894^b^	–
Gender ratio (male/total, %)	50.00	47.12	0.821^a^	–
Onset age (median, IQR)	60 (52–64)	60 (56–63)	0.734^b^	–
Disease duration (median, IQR)	5 (2–10)	4 (2–6)	0.367^b^	–
Expansion (median, IQR)	115.5 (100.5–138.0)	119.0 (96.0–150.3)	0.883^b^	0.923^c^
Total SCOPA-AUT score (median, IQR)	9.50 (3.00–16.00)	19.00 (15.00–24.00)	<0.001^b^	0.002^c^
Digestive domain (median, IQR)	2.00 (0.00–4.75)	2.50 (0.00–4.50)	0.912^b^	0.817^c^
Swallowing/choking (median, IQR)	0.00 (0.00–1.00)	0.00 (0.00–1.00)	0.382^b^	0.359^c^
Sialorrhea (median, IQR)	0.00 (0.00–0.00)	0.00 (0.00–1.00)	0.293^b^	0.414^c^
Dysphagia (median, IQR)	0.00 (0.00–0.00)	0.00 (0.00–0.00)	0.190^b^	0.183^c^
Early abdominal fullness (median, IQR)	0.00 (0.00–0.00)	0.00 (0.00–1.00)	0.619^b^	0.855^c^
Constipation (median, IQR)	0.00 (0.00–1.00)	0.00 (0.00–1.25)	0.627^b^	0.737^c^
Straining for defecation (median, IQR)	0.00 (0.00–1.00)	0.00 (0.00–2.00)	0.703^b^	0.564^c^
Fecal incontinence (median, IQR)	0.00 (0.00–0.00)	0.00 (0.00–0.00)	0.115^b^	0.177^c^
Urinary domain (median, IQR)	3.00 (1.00–5.75)	11.50 (7.00–17.25)	<0.001^b^	<0.001^c^
Urgency (median, IQR)	0.00 (0.00–1.00)	2.00 (1.00–3.00)	<0.001^b^	<0.001^c^
Urinary incontinence (median, IQR)	0.00 (0.00–1.00)	1.50 (0.00–3.00)	0.09^b^	0.003^c^
Incomplete emptying of bladder (median, IQR)	0.00 (0.00–2.00)	2.00 (1.00–3.00)	<0.001^b^	<0.001^c^
Weak stream of urine (median, IQR)	0.00 (0.00–1.75)	2.00 (0.75–3.00)	<0.001^b^	<0.001^c^
Frequency of urinatior (median, IQR)	0.00 (0.00–1.00)	2.00 (1.00–3.00)	<0.001^b^	<0.001^c^
Nocturia (median, IQR)	1.00 (0.00–2.00)	3.00 (2.00–3.00)	<0.001^b^	<0.001^c^
Cardiovascular domain (median, IQR)	0.00 (0.00–2.00)	0.00 (0.00–2.00)	0.848^b^	0.609^c^
Lightheaded (standing up) (median, IQR)	0.00 (0.00–1.00)	0.00 (0.00–1.00)	0.416^b^	0.439^c^
Lightheaded (standing for some time) (median, IQR)	0.00 (0.00–1.00)	0.00 (0.00–1.00)	0.754^b^	0.903^c^
Syncope (median, IQR)	0.00 (0.00–0.00)	0.00 (0.00–0.00)	0.372^b^	0.574^c^
Thermoregulatory domain (median, IQR)	0.00 (0.00–2.00)	1.50 (0.00–3.00)	0.321^b^	0.326^c^
Hyperhidrosis (day) (median, IQR)	0.00 (0.00–0.00)	0.00 (0.00–1.00)	0.455^b^	0.406^c^
Hyperhidrosis (night) (median, IQR)	0.00 (0.00–0.00)	0.00 (0.00–0.00)	0.723^b^	0.677^c^
Cold intolerance (median, IQR)	0.00 (0.00–0.00)	0.00 (0.00–2.00)	0.134^b^	0.077^c^
Heat intolerance (median, IQR)	0.00 (0.00–0.00)	0.00 (0.00–0.25)	0.872^b^	0.818^c^
Pupillomotor (median, IQR)	0.00 (0.00–0.00)	0.00 (0.00–1.25)	0.047^b^	0.064^c^
Sexual domain (median, IQR)	0.00 (0.00–0.00)	0.00 (0.00–0.50)	0.110^b^	0.063^c^
Sexual domain: men (median, IQR)	0.00 (0.00–0.00)	0.00 (0.00–6.00)	0.032^b^	0.026^d^
Erection problem (median, IQR)	0.00 (0.00–0.00)	0.00 (0.00–3.00)	0.038^b^	0.051^d^
Ejaculation problem (median, IQR)	0.00 (0.00–0.00)	0.00 (0.00–3.00)	0.023^b^	0.033^d^
Sexual domain: women (median, IQR)	0.00 (0.00–0.00)	0.00 (0.00–0.00)	0.477^b^	0.517^d^
Vaginal lubrication dysfunction (median, IQR)	0.00 (0.00–0.00)	0.00 (0.00–0.00)	0.476^b^	0.484^d^
Problem with orgasm (median, IQR)	0.00 (0.00–0.00)	0.00 (0.00–0.00)	0.564^b^	0.589^d^

Onset-with-AutD: patients present with AutD as their initial symptoms, onset-without-AutD cases: patients did not present with AutD as their initial symptoms.

a Chi-square test.

b Mann–Whitney test.

c Adjusted by sex and age.

d Adjusted age.

By comparing the differences in autonomic symptoms between the two groups, we found that the symptoms of urinary urgency, urinary incontinence, incomplete emptying of bladder, weak stream of urine, frequency of urination, nocturia, and ejaculation problems were more frequently seen in the subgroup that began with autonomic dysfunction (*p* < 0.05, adjusted *p* < 0.05) (Table 5). These results suggest that patients with an early onset of unexplained urinary symptoms or male sexual dysfunction should be considered for NIID diagnosis.

## Discussion

NIID is a rare disease with variable clinical manifestations, and further investigation is required to explore the clinical spectrum of this disease. In this study, we recruited a large cohort of patients with NIID and performed comprehensive clinical evaluation of autonomic dysfunction. We found that patients with NIID usually presented with autonomic symptoms, particularly in the urinary, gastrointestinal, thermoregulatory, and cardiovascular domains.

The most prominent complaints in patients with NIID were urinary system and gastrointestinal tract dysfunction, including nocturia, incomplete emptying of bladder, constipation, straining for defecation, and frequency of urination. The rate of these symptoms differed from other reported studies (3). The prevelance of miosis in this study was lower than that in the Japanese cohort, possibly because our patients were diagnosed at an early stage of the disease through genetic testing. However, the sample size and application of systematic questionnaires could also lead to these differences. Compared with age- and sex-matched controls, patients with NIID experienced significantly more problems with urinary, gastrointestinal, thermoregulatory, cardiovascular, pupillomotor, and male sexual dyfunction. However, there was no significant difference in female sexual dysfunction, which may be related to the fact that females may tend to deny sexual problems, and the statistical analysis of the sexual domain may be biased.

Compared with previous study (20), patients with MSA had higher scores in total SCOPA-AUT, urinary domain and sexual domain, while the patients with NIID of this study had higher score in the digestive system. These finding suggest that the severity and involved domain of autonomic dysfunction differed between NIID and other neurodegenerative diseases.

The manifestations of AutD in NIID have been reported in many cases (12, 27, 28), and the mechanism may be related to the formation of neuronal intranuclear inclusions in the autonomic nerves or the loss of autonomic neurons. Sone et al. (10) reported that eosinophilic inclusion bodies were widespread, especially in sympathetic and myenteric ganglion neurons, dorsal root ganglion neurons, and spinal motor neurons in the autopsy of two patients with NIID with peripheral neuropathy and AutD. In an autopsy of a 21 years-old female patient, Sung et al. (29) found that the Onuf’s nucleus and the intermediolateral nucleus of sacral autonomic neurons were largely lost. Two studies also revealed that many intranuclear inclusions were located in the hypothalamus and autonomic nerve centers (10, 30).

In the present study, we first applied the SCOPA-AUT questionnaire to evaluate autonomic symptoms in patients with NIID. The SCOPA-AUT is comprehensive and easy to operate. The AUCs of SCOPA-AUT were >0.7 in total score, gastrointestinal domain, and urinary domain, suggesting that the SCOPA-AUT could be used as a diagnostic and quantitative tool for autonomic dysfunction in NIID.

We also discovered that AutD in patients with NIID was positively correlated with disease duration and age, indicating that autonomic dysfunction in patients with NIID gradually increased with disease progression. Simultaneously, the study revealed that AutD was associated with impaired activities of daily living and mental symptoms in patients with NIID. This may be due to autonomic nervous dysfunctions, such as urinary incontinence and dysphagia, which usually lead to inconvenience in daily life and affect daily function. Although it has been reported that psychiatric symptoms, such as depression, have an important impact on autonomic problems in patients with PD (31), this study was only a cross-sectional study, and we were not able to draw a causal relationship between the symptoms through this result.

Additionally, we found that 18/122 (14.75%) patients with NIID with AutD as onset symptoms showed significantly higher scores in the total SCOPA-AUT, urinary domain, and sexual domain (*p* < 0.05), compared with those of patients with NIID with the onset without AutD, and there were no significant differences in age and disease duration between these two groups. This suggests that onset-with-AutD NIID may suffer more severe autonomic dysfunction than those of onset-without-AutD NIID. We also observed that the symptoms of urinary urgency, urinary incontinence, incomplete emptying of bladder, weak stream of urine, frequency of urination, nocturia, and ejaculation problems, were more frequently seen in the onset-with-AutD subgroup than in the onset-without-AutD subgroup (all *p* < 0.05). Eighteen patients with NIID started with urinary symptoms of unknown origin and the relevant symptomatic treatment was ineffective. In severe cases, cystostomy or long-term indwelling catheter insertion was performed before the appearance of other symptoms. These findings indicate that autonomic symptoms may occur before the onset of other NIID symptoms, as previously reported (14). The high prevalence of AutD suggests that NIID should be considered in patients with AutD, especially in those with unexplained AutD alone.

This study had limitations, as it only referred to autonomic nervous dysfunction in patients with adult-onset NIID, and further longitudinal studies are needed to elucidate its mechanism and changes during disease duration. This study also did not combine the corresponding autonomic nerve function examination, due to the fact that some patients were in an acute stage of the disease and could not complete objective test for autonomic dysfunction such as Quantitative Sudomotor Axon Reflex Tests (ex: QSART, QSWEAT) or urodynamic studies. In addition, many patients had undergone cystostomy or long-term indwelling catheter insertion in the early stage of the disease, which made it difficult to carry out relevant auxiliary examinations. Therefore, only 14 patients underwent bladder residual urine volume examination in this study, of which 10 patients showed increased bladder residual urine volume. For patients with NIID, objective examination of autonomic nervous function is necessary, and further studies with larger sample size are needed to explore the autonomic dysfunction in NIID. Finally, SCOPA-AUT had its own limitation in assessing autonomic dysfunction of NIID. For instance, episodic vomiting, which was frequently observed in many patients with NIID (3, 13), was not included in SCOPA-AUT.

## Conclusion

In conclusion, SCOPA-AUT can be used for evaluation of autonomic dysfunction in NIID. The high prevalence of AutD in patients with NIID suggests that NIID diagnosis should be considered in patients with AutD, especially in those with unexplained AutD alone. In addition, AutD in patients with NIID is related to age, disease duration, impairment of daily living ability, and psychiatric symptoms.

## Data availability statement

The original contributions presented in the study are included in the article/supplementary material, further inquiries can be directed to the corresponding author.

## Ethics statement

The studies involving human participants were reviewed and approved by the Ethics Committee of the Xiangya Hospital, Central South University. The patients/participants provided their written informed consent to participate in this study.

## Author contributions

LS and BT original idea and revision of draft. LZ draft of the paper and participants data collection. YT draft of the paper and statistical analyses. SZ statistical analyses. BJ, XL, and YZ patients clinical diagnosis. QX, JX, and RD gene mutation detection. All authors contributed to the article and approved the submitted version.

## Funding

This study supported by the National Key R&D Program of China (nos. 2020YFC2008500 and 2022ZD0213700), the National Natural Science Foundation of China (nos. 81971029, 81671075 and 82071216, 81901171), and Hunan Innovative Province Construction Project (no. 2019SK2335).

## Conflict of interest

The authors declare that the research was conducted in the absence of any commercial or financial relationships that could be construed as a potential conflict of interest.

## Publisher’s note

All claims expressed in this article are solely those of the authors and do not necessarily represent those of their affiliated organizations, or those of the publisher, the editors and the reviewers. Any product that may be evaluated in this article, or claim that may be made by its manufacturer, is not guaranteed or endorsed by the publisher.
